# Coping with potential bi‐parental inbreeding: limited pollen and seed dispersal and large genets in the dioecious marine angiosperm *Thalassia testudinum*

**DOI:** 10.1002/ece3.2309

**Published:** 2016-07-13

**Authors:** Brigitta Ine Van Tussenbroek, Tania Valdivia‐Carrillo, Irene Teresa Rodríguez‐Virgen, Sylvia Nashieli Marisela Sanabria‐Alcaraz, Karina Jiménez‐Durán, Kor Jent Van Dijk, Guadalupe Judith Marquez‐Guzmán

**Affiliations:** ^1^ Instituto de Ciencias del Mar y LimnologíaUnidad Académica Sistemas Arrecifales‐Puerto MorelosUniversidad Nacional Autónoma de MéxicoProlongación Niños Héroes S/NPuerto MorelosQuintana RooMéxico; ^2^ Facultad de CienciasLaboratorio de Desarrollo de PlantasCiudad UniversitariaUniversidad Nacional Autónoma de MéxicoCoyoacanDistrito FederalMéxico; ^3^ Present address: Department of Life and Health SciencesUniversity of North Texas at DallasDallasTexas; ^4^ Present address: School of Biological ScienceUniversity of AdelaideAdelaideSouth Australia5005Australia

**Keywords:** Clonal growth, genetic neighborhood, genetic structure, hydrodynamics, hydrophily, mating system, microsatellite markers, seagrass

## Abstract

The high prevalence of dioecy in marine angiosperms or seagrasses (>50% of all species) is thought to enforce cross‐fertilization. However, seagrasses are clonal plants, and they may still be subject to sibling‐mating or bi‐parental inbreeding if the genetic neighborhood is smaller than the size of the genets. We tested this by determining the genetic neighborhoods of the dioecious seagrass *Thalassia testudinum* at two sites (Back‐Reef and Mid‐Lagoon) in Puerto Morelos Reef Lagoon, Mexico, by measuring dispersal of pollen and seeds in situ, and by fine‐scale spatial autocorrelation analysis with eight polymorphic microsatellite DNA markers. Prevalence of inbreeding was verified by estimating pairwise kinship coefficients; and by analysing the genotypes of seedlings grown from seeds in mesocosms. Average dispersal of pollen was 0.3–1.6 m (max. 4.8 m) and of seeds was 0.3–0.4 m (max. 1.8 m), resulting in a neighborhood area of 7.4 m^2^ (range 3.4–11.4 m^2^) at Back‐Reef and 1.9 (range 1.87–1.92 m^2^) at Mid‐Lagoon. Neighborhood area (Na) derived from spatial autocorrelation was 0.1–20.5 m^2^ at Back‐Reef and 0.1–16.9 m^2^ at Mid‐Lagoon. Maximal extensions of the genets, in 19 × 30 m plots, were 19.2 m (median 7.5 m) and 10.8 m (median 4.8 m) at Back‐Reef and Mid‐Lagoon. There was no indication of deficit or excess of heterozygotes nor were coefficients of inbreeding (*F*_IS_) significant. The seedlings did not show statistically significant deficit of heterozygotes (except for 1 locus at Back‐Reef). Contrary to our expectations, we did not find evidence of bi‐parental inbreeding in this dioecious seagrass with large genets but small genetic neighborhoods. Proposed mechanisms to avoid bi‐parental inbreeding are possible selection against homozygotes during fecundation or ovule development. Additionally, the genets grew highly dispersed (aggregation index Ac was 0.09 and 0.10 for Back‐Reef and Mid‐Lagoon, respectively); such highly dispersed guerrilla‐like clonal growth form likely increases the probability of crossing between different potentially unrelated genets.

## Introduction

All species of marine angiosperms (also named seagrasses) depend on water and its movement for dispersal of pollen and seeds. Distances of dispersal of pollen transported under water or at the water surface are generally limited, in the order of meters (Cox and Tomlinson 1988; Ruckelshaus 1996; Verduin et al. 1996; Smith 2000; Ackerman 2002; McMahon et al. 2014), although the seeds transported by water may disperse much farther (McMahon et al. 2014). Many seagrasses have dual modes of seed dispersal; the first involves floating fragments such as flowering branches or fruits, which are dispersed over long distances (10^0^–10^3^ km) by superficial currents (Orth et al. 2006; Van Dijk et al. 2009; Kendrick et al. 2012). The second mode is much more common and consists of local dispersal on the sea bottom as the seeds are negatively buoyant (McMahon et al. 2014). Sea bottom micro‐topographic features such as sand‐ripples and benthic fauna often enhance seed retention, thus dispersal distances are in the order of 1–10 m (Ruckelshaus 1996; Lacap et al. 2002), although Manley et al. (2015) reported mean local seed dispersal distances of an order of magnitude higher for *Zostera marina*. The dispersal potential of seeds and pollen establishes the spatial extent of local gene flow and determines the size of local genetic neighborhoods (Wright 1969; Crawford 1984). Although studies on direct measurements of dispersal of seagrasses have recently increased (see Ackerman 2006 and Orth et al. 2006 for reviews on pollen and seed dispersal, respectively), only Ruckelshaus (1996) has attempted to estimate the neighborhood size of a seagrass (*Z. marina*) based on direct measures of pollen and seed dispersal.

The size of the genetic neighborhood can also be derived indirectly from the local genetic structure, quantified by fine‐scale spatial autocorrelation of the ramets and genets (Heywood 1991). Indirect fine‐scale genetic estimates of neighborhood areas have been estimated with microsatellites for the monoecious (male and female flowers on the same plant) *Z. marina* (Reusch et al. 1999a; Hämmerli and Reusch 2003; Billingham et al. 2007), *Z. noltei* (Zipperle et al. 2011), *Posidonia oceanica* (Migliaccio et al. 2005; Diaz‐Almela et al. 2007), *P. australis* (Sinclair et al. 2014), and the dioecious (male and female flowers on different plants) *Cymodocea nodosa* (Alberto et al. 2005; Ruggiero et al. 2005a). The above mentioned studies reported local gene dispersal in the order of meters, but they also detected that local genetic structures are influenced by the clonal growth of the seagrasses.

Extensive clonal growth may have a direct effect in the reproductive success and evolution of mating systems (Charpentier 2002), because it can interfere with the flow of pollen between genets (groups of genetically identical individuals derived by asexual/clonal reproduction from the same zygotes). Inbreeding among clonal plants may be common depending on spatial arrangements of the genets, mating systems, and dispersal strategies. Geitonogamy (transfer of pollen between genetically identical flowers) is frequent in some terrestrial clonal species (Charpentier 2002) and has been reported in both monoecious (Reusch 2001; Ruggiero et al. 2005b; Zipperle et al. 2011) and hermaphrodite seagrasses with male and female structures on the same flower (Les 1988; Waycott and Sampson 1997; Sandmeier et al. 1999; Migliaccio et al. 2005). This sexual reproductive strategy of self‐fertilization may negatively affect fitness (Charpentier 2002); and selfed offspring of the seagrass *Z. marina* exhibited lower fitness than outcrossed individuals (Reusch 2001). It has been suggested that the high prevalence of dioecy in seagrasses (53% of all species) may have evolved to enforce cross‐fertilization (Les 1988; Reusch 2001). However, mates of dioecious clonal species may still be related through common ancestry within a population resulting in sib‐mating (Les 1988). The effects of bi‐parental inbreeding (mating between genetically related individuals) through sib‐mating, on fitness may not be as obvious as that of selfed offspring, but total fitness of the population may be compromised by lower seed germination success (Richards 2000), progeny size (Waser and Price 1994), chance of progeny survival (Heywood 1993; Waser and Price 1994), or maternal fecundity (Ashman 1992; Nason and Ellstrand 1995).

The frequency of occurrence of bi‐parental inbreeding in dioecious clonal plants will depend on the dispersal potential of the sexual propagules in combination with their clonal growth strategies, which determine the size (extension) of the genets and the spatial distribution of their ramets. The species under study, *Thalassia testudinum*, is a climax species that forms large genets (Van Dijk and Van Tussenbroek 2010). It is dioecious with likely limited pollen dispersal (Van Tussenbroek and Muhlia Montero 2013) and a dual seed dispersal strategy (Van Dijk et al. 2009). We expect that local seed and pollen dispersal distances are less that the extension of the genets, and that therefore this species suffers from inbreeding through sibling‐mating. To verify this, we compare direct (pollen and seed dispersal) and indirect (spatial autocorrelation) estimates of the spatial size of the genetic neighborhood of *T. testudinum* at two sites in a tropical reef lagoon (Mexican Caribbean) with the following goals: (1) To deduce the potential for bi‐parental inbreeding from estimates of gene dispersal and size of the genets; (2) To verify the prevalence of inbreeding by estimating pairwise kinship coefficients, and by testing seedlings for deficiency of heterozygotes.

## Materials and Methods

### Study species

*Thalassia testudinum* dominates extensive seagrass communities along the tropical and subtropical coasts of the Western Atlantic, where it provides essential ecosystem services such as provision of food and shelter to fauna, coastal protection, and carbon sequestration. It is a perennial clonal plant with varying abundance of sexual reproductive structures, depending on local conditions (Van Dijk and Van Tussenbroek 2010). Its flowers are situated 1–2 cm above the sea floor. Male flowers occur in clusters of one to five flowers (usually two or three) and have copious pollen production [in the order of 10^5^ grains per flower (Van Tussenbroek et al. 2009)]. Pollen grains (diam 52–56 *μ*m) are released in strands or masses of neutrally to slightly negatively buoyant mucilage (Cox and Tomlinson 1988; Van Tussenbroek et al. 2009). Female flowers usually occur singly with an inferior ovary below sediment level that develops into a relatively large fruit (diam 2.0–2.5 cm) with 1–6 (usually 2–4) seeds.

**Figure 1:**
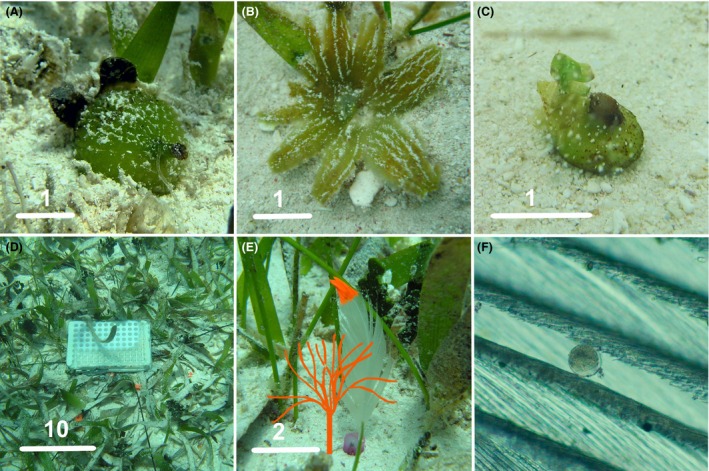
*Thalassia testudinum*. (A) Mature fruit before dehiscence (bar = 1 cm), (B) Open fruit (bar = 1 cm), (C) Recently released seed (bar = 1 cm), (D) Device for release of pollen in the Back‐Reef meadow (bar = 10 cm), (E) Feather used to trap pollen; together with a drawn female flower (bar = 2 cm), (F) Pollen grain trapped in the barbs of the feather; the diameter of pollen grain ~ 55 *μ*m.

In the study area, flowering generally occurs from March until May and the fruits ripen in September–October (Van Tussenbroek 1994). Small proportions of fruits (10–15% detach from the pedicel and float to the surface (Van Dijk et al. 2009), providing a mechanism of migration out of the donor bed. The majority of the fruits open in situ when still attached to the mother plant; providing a mechanism of seed retention (Fig. 1A–C). The negatively buoyant seeds (max diam 0.8–1.5 cm and 22–60 mg wet weight; B. I. Van Tussenbroek, unpubl. data) lack hard seed coats and do not pass through a stage of dormancy (Orpurt and Boral 1964).

### Study site

This study was conducted in the tropical Puerto Morelos Reef Lagoon, Caribbean Sea, Mexico (20°51′ N; 86°55′ W), which is bordered on the seaward site by a fringing reef between 500 and 1800 m from the coast. Terrestrial influence is virtually absent because of the lack of surface rivers and salinity is 35.7‰. Monthly mean surface water temperatures (1993–2005) varied between 25.1°C in the winter and 29.9°C in the summer (Rodríguez‐Martínez et al. 2010). Average currents varied between 10 and 20 cm sec^−1^ with a predominant S to N direction parallel to the coast, although current may invert at times (Coronado et al. 2007). A well‐developed seagrass community with dominance of *Thalassia testudinum* covers the coarse calcareous sands in this reef lagoon. Flowering frequency of this seagrass varies throughout the reef lagoon, generally being lower in near‐coastal and mid‐lagoon areas and higher near the reef.

The two main study sites in the reef lagoon are separated by about 1.2 km, both forming part of the same continuous growing meadow. These sites were identified as Back‐Reef (20°51′51.2′′ N, 86°51′29.5′′ W, depth ~3.0 m) and Mid‐Lagoon (20°51′39.8′′ N, 86°52′06.5′′ W, depth ~3.5 m) at a distance of approximately 100 and 900 m from the reef crest, respectively. Seed dispersal was determined at an additional mid‐lagoon site (Mid‐Lagoon 2, 20°52′14.6′′ N, 86°51′27.5′′ W, depth 3–4 m, distance from reef crest ~400 m).

### Dispersal of seeds and pollen

#### Seed dispersal

**Figure 2:**
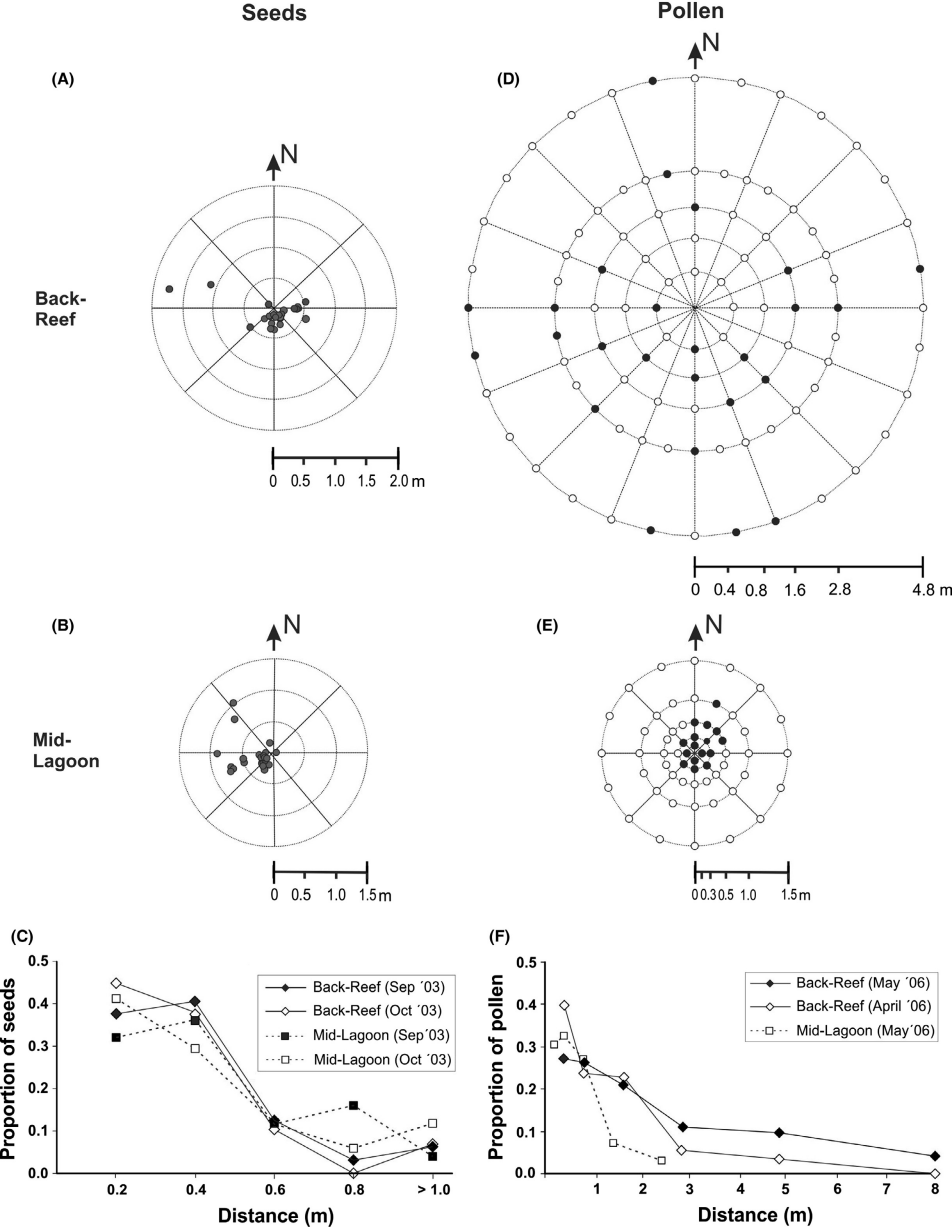
Dispersal of seeds of *Thalassia testudinum* at (A) Back‐Reef, 8–10 October 2003 (N 29), and (B) Mid‐Lagoon, 23–26 September 2003 (N 25). Each dot represents a seed. (C) Distribution of dispersal distances of the seeds from experiments conducted in September – October 2003 (see Table 1 for additional information). (D, E) Dispersal of pollen at Back‐Reef (10 June 2005) and Mid‐Lagoon (29 April 2005), respectively. Each dot represents a trap (feather); the dark dots represent traps with pollen. (F) Distribution of dispersal distances of the seeds from experiments conducted in April and May 2006 (see Table 2 for additional information).

Four field simulations of seed dispersal were performed in September and October 2003 (see Table 1). The seeds were obtained from ripe fruits and painted with a bright orange spray. The point of release of the seeds was marked with a brightly painted galvanized nail pushed into the sand until only the upper surface of the head was visible. Thirty seeds were deposited on the sea bottom per trial. To avoid disturbance by water‐waves created by divers, release of seeds was performed by placing a 1 m long PVC tube (diam 5 cm) 2–5 cm above the point of release. After 2–3 days (when the seeds had formed root hairs), the painted seeds were spotted while SCUBA diving 1–1.5 m above the sea floor and their positions were marked using a small weight with a ~1 m long cord and small floater. When all visible seeds were located, the distance and position from the initial point of release were determined for each seed with a metric tape and compass (10° precision), respectively (Fig. 2). After establishing the position of all visible seeds, a search was performed for buried seeds or those hidden under small rocks, corals or algae in a perimeter of ~3 m from the point of release. The rate of recovery was generally high (Table 1).

**Table 1:** *Thalassia testudinum*. Seed dispersal variables for Puerto Morelos Reef Lagoon, Mexico (2003). Thirty seeds were released per trial and trials lasted 2–3 days

Site	Date	*N*	Mean (m)	Max (m)	Hodjes‐Ajne, “m”	Main direction (°)	*V*_0_ (m)	*K*	AF	*N*_aSeed_ (m^2^)
Back‐Reef	9–11 Sep	29	0.34	1.74	3***	90–160	0.056	11.18***	3.66	0.64
Back‐Reef	8–10 Oct	29	0.39	1.80	3***	90–180	0.054	12.44***	3.66	0.62
Mid‐Lagoon	23–26 Sep	17	0.37	0.91	5^NS^	NA	0.035	NS	4	0.44
Mid‐Lagoon2	23–26 Sep	25	0.36	1.03	4**	230–250	0.039	NS	4	0.48

*N*: number of recovered seeds, Mean: mean dispersal distance, Max: maximal dispersal distance, Hodjes‐Ajne: “m”‐statistic of the test for circular uniformity test for circular uniformity, Main direction: range of direction of displacement of >50% of total number of seeds, *V*_0_: axial variance, *K*: kurtosis (significance was determined with paired t‐test), AF: approximate area correction factor (Wright 1969), *N*_aSeed_: neighborhood area for seeds. ***P < 0.001, **P < 0.01, *P < 0.05, NS: not significant at *α *= 0.05, NA: not applicable.

A Hodges–Ajne statistical was used to test whether the seeds were uniformly distributed in a circle (the Hodges–Ajne test only considers the direction of displacement of each point and does not assume a specific distribution, Zar 1999). The distribution of seed dispersal distances was determined as the proportion of total number of recovered seeds at each distance.

#### Pollen dispersal

Pollen dispersal distances were determined by means of pollen traps, which consisted of small recently plucked chicken feathers with the vane trimmed to a width of 3–4 cm and a total height of 8 cm (Fig. 1E). The lower part of the shaft was covered with epoxy putty, which served for anchorage in the sand. The tip of the feather was marked with bright acrylic paint and a number. The pollen was retrieved from male floral buds collected a day before the experiment. The flowers were placed in a Petri dish with seawater where pollen was released overnight. The pollen‐mucilage mass was placed in a vial with a small quantity of seawater for transport to the experiment site. A pollen liberation device consisted of a weighted 96‐well 400 *μ*L microtiter plate (total volume ~37 cm^3^) and a weighted lid lined on the inside with a rubber seal to warrant perfect sealing. The pollen was placed in the wells just before release in the 96 wells, making sure no air was trapped under the lid. The assembly was then placed on the bottom of the sea floor at the release point (Fig. 1D). A diver positioned well above the experimental area lifted the lid cautiously using a long cord to minimize interference. The trials were realized at mid‐day to avoid “contamination” with pollen from other male flowers in the seagrass bed. Male flowers of *T. testudinum* open only at dusk and pollen release is complete after a couple of hours (Van Tussenbroek et al. 2009).

Initial field trials in 2005 served to find the maximal dispersal distance at each site, to design subsequent experiments in April and May 2006. Feathers were placed in circles around the point of pollen release at distance intervals (Fig. 2). The distance intervals between the circles with pollen traps followed a Fibonacci sequence; this was the best compromise between relevant resolution of the sampling frequency and the extension of the “search” area. The experimental area was prepared in advance by placing galvanized nails in the sediment at each trap position. The main cardinal axes were fixed using a submergible compass. The numbered feathers were placed in predetermined positions with the concave site of the vane facing the point of release. The devise of pollen release filled with the pollen‐mucilage mass mixed with seawater was placed in the center of the circle and pollen was released promptly. This procedure was repeated three times with intervals of 10–15 min. After 30 min of the last release, the traps were collected, starting with the peripheral ones, to avoid wave disturbance by divers. To prevent detachment of pollen, each trap was covered with a 50 mL Falcon tube and gently released from the sand before closing the tube with a cap. The tubes were transported to the laboratory and stored at 4°C until examination. Each trap was examined using an optical microscope. The pollen grains were clearly visible between the barbs of the feather (Fig. 1F). During the 2005 trials, absence or presence of grains in traps was recorded, whereas in 2006 all pollen grains were counted on each side of the trap. A control experiment was also run to account for nonexperimental pollen capture at the two sites and consisted of 50 feathers placed in the seagrass beds during the same time of the day at times of abundant flowering (May 2005).

During the experiments in 2005, water velocity was measured with an Argonaut ADV^®^ (SonTek, San Diego, CA, USA), which measured single point velocities in a 0.25 cc sampling volume at intervals of 5 sec for 1 h. This Doppler velocity meter was located at ~50 m distance from the experimental area in the seagrass bed. The ADV was placed in a small manually cleared patch and the sensor was positioned to measure velocities at ~2 cm above the sea bottom which corresponded with the height of natural and experimental pollen release.

The distribution of pollen dispersal was derived from the experiments in 2006 (Table 3) and was the portion of the total number of trapped grains at each distance, with a correction of sampling effort per distance interval (no traps/circumference). A Hodges‐Ajne statistical test for circular distribution was applied to test whether the pollen grains were uniformly distributed in a circle (Zar 1999).

### Calculation of neighborhood area (*N*_a_) based on seed‐, and pollen dispersal

Neighborhood area based on the dispersal of pollen and seeds was determined for each sampling site (Back‐Reef and Mid‐Lagoon) as $N_{a}=\text{AF}\pi \left(\sigma_{p}^{2}/2+\sigma_{s}^{2}\right)$ . Where AF is an aerial correction factor for kurtosis of the distribution curve of dispersal distances (approximation following Wright 1969: if there is no kurtosis AF = 4) and $\sigma_{p}^{2}$ and $\sigma_{s}^{2}$ are the one‐way or axial variance of pollen and seed dispersal distances, respectively, measured along a single axis from the source.

### Population genetics

**Figure 3:**
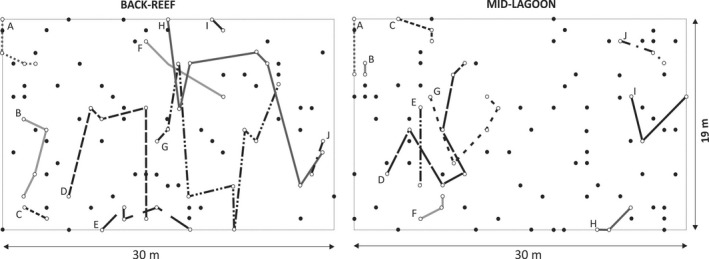
Spatial distribution of ramets of *Thalassia testudinum* at the two sampling sites in the Puerto Morelos Reef Lagoon. The positions of the ramets are indicated by circles: open circles connected with lines represent ramets belonging to the same genet and closed circles represent unique genotypes. Distinct genets are indicated by letters and different line patterns.

Fine‐scale spatial genetic structure (SGS) of *T. testudinum* at Mid‐Lagoon and Back‐Reef sites was determined in sample areas of 570 m^2^ (19 × 30 m). Within this area 620 grid points were selected at 1 m intervals and 100 coordinates (sampling points) were randomly selected (Fig. 3). Sampling was performed during March and April 2008. A grid was laid out with strings and nails. At each sampling point, the nearest foliar shoot was collected by cutting the vertical rhizome below sediment level. The mean shoot density at Mid‐Lagoon and Back‐Reef was 547 and 693 shoots per m^2^, respectively; thus, samples comprised <0.03% of total number of ramets at the sample areas at both sites. Leaf‐sheath tissues were separated and preserved in silica gel as described by Van Dijk et al. (2007).

In the laboratory, DNA was extracted from the tissue samples after pulverizing 10–20 mg of dried sheath tissue through ultra‐agitation with a Mini Beadbeater^™^ (Biospec Products, Bartlesville, OK, USA). DNA extractions were performed using DNeasy^®^ Plant Minikit (QIAGEN, Venlo, the Netherlands), following the manufacturer's instructions. DNA concentrations of the extractions were determined using a NanoDrop 2000 spectrophotometer (Thermo Scientific, Waltham, MA, USA) and DNA samples were diluted to 15 ng *μ*L^−1^ for amplification. Polymerase Chain Reactions (PCR) were performed using eight microsatellite loci: TTMS‐GA6, TTMS‐GA8, TTMS‐TGA39, TTMS‐TCT58, TTMS‐GGT59, TTMS‐GT112, TTMS‐GT77, and TTMS‐Th1MS (Van Dijk et al. 2007) where the forward primers where modified by adding the VIC, NED, 6FAM, and PET fluorescent dyes to allow for multiplexing. Reaction conditions were followed as described by Van Dijk et al. (2007) with modifications (Table S3). The amplifications were sent for genotyping on an ABI 3730xl Genetic Analyzer (Applied Biosystems, Inc., Foster City, CA, USA) to the Roy Carver Biotechnology Center at the University of Illinois. LIZ 500^®^ (Applied Biosystems Inc.) was used as size standard. Alleles for each sample were scored using GeneMapper^®^ software v4.0 (Applied Biosystems Inc.). The obtained genotypes were analyzed with Genclone v2.0 (Arnaud‐Haond and Belkhir 2007) to verify if small genotypic differences were due to scoring errors or to confirm they were real.

### Clone identification

Genclone v2.0 (Arnaud‐Haond and Belkhir 2007) was used to identify the samples that shared the same multi locus genotypes (MLGs). To assess the likelihood that identical MLGs resulted from sampling of the same clone/genet at two different spatial coordinates or that these were identical genotypes resulting from two distinct sexual reproduction events *p*_sex(f)_ was calculated. This probability illustrates the likelihood of a repeated genotype being of sexual origin given the allelic diversity of each population, taking into account the estimated inbreeding coefficient *F*_IS_ (Alberto et al. 2005; Arnaud‐Haond et al. 2007). After establishing the number of genets in each population the clonal richness for each was calculated as *R *= (*G*−1)/(*N*−1), where *N* is the number of genotyped samples and *G* the number of MLGs in the population (Dorken and Eckert 2001). To describe if identical clone mates were randomly distributed within the populations or if these tended to grow near each other, the aggregation index A_C_ was also calculated with Genclone v2.0 (Arnaud‐Haond and Belkhir 2007) according to equation in Arnaud‐Haond et al. (2007).

For following population genetic inferences all replicate samples for each MLG were eliminated from each population, thus, each MLG was represented only once. Basic genetic descriptors such as allele frequencies, observed heterozygosity (*H*_O_), expected heterozygosity or Nei's gene diversity *H*_E_ (Nei 1973), average number of alleles (*A*), and the inbreeding coefficient (*F*_IS_) were calculated with ARLEQUIN v3.5.1.2 (Excoffier and Lischer 2010) for each site. GENEPOP v4.0 (Rousset 2008) was used to calculate deviations from Hardy–Weinberg (HW) equilibrium for each population using the global heterozygosity test for excess and deficit and to test for linkage disequilibrium between loci.

#### Spatial population genetic structure

To address the effects of clonal and sexual reproduction on spatial genetic structure (SGS), we calculated the kinship coefficient *F*_IJ_ with the program SPAGeDi v1.4 (Hardy and Vekemans 2002), where *F*_IJ_ is the probability of a pair of samples being genetically identical based on their alleles (Ritland 1996). A spatial autocorrelation analysis was performed using *F*_IJ_ and this was plotted against the pairwise spatial distance between ramets (all samples included) and genets to infer the clonal subrange (Arnaud‐Haond and Belkhir 2007). For the genet level analysis, distinct genotypes were included only once based on central coordinates (most parsimonious position of the clone birthplace random coordinates) and random coordinates (a random ramet coordinate representative of the clone).

The average kinship coefficients were estimated for 19 distance classes with 1.5‐m intervals (total range 0–30 m) with a final (20th) class of 30–35.5 m. The first size class 0–1.5 m allows for the inclusion of most near‐neighbor pairs.

Pairwise kinship estimates were regressed on the spatial distance to estimate the slope of the linear regression (*b*_log_). For each sampling locality, spatial coordinates were randomly permuted 10,000 times among individuals, for each distance class, in order to test if the observed mean kinship values were different from those expected under random distribution of genotypes. The slope of the corresponding regressions was used to estimate gene dispersal distances.

The rate of decrease in pairwise kinship with distance, *Sp* = −*b*_log_/(1−*F*_IJ(1.5 m)_), and neighborhood size, *Nb* = −(1 − *F*_IJ(1.5 m)_)/b_log_, were estimated, from the *b*_log_ of the regression of the autocorrelation analysis (following Vekemans and Hardy 2004), where *F*_(1.5 m)_ is the average *F*_IJ_ between individuals belonging to the first distance class.

Kinship is expected to decrease linearly with the spatial distance for a restricted range (*σ* to 20*σ*, where *σ* is the axial standard deviation of gene dispersal distances; Vekemans and Hardy 2004; Volis et al. 2010). Sigma (*σ*) was determined for each sampling locality as the standard deviation of gene dispersal distance (*σ *= (Nb/(4*π*D_e_))^0.5^ as described in Volis et al. (2010) where D_e_ is the effective population density derived here from ramet (shoot) density multiplied with clonal richness (R). Finally, neighborhood area (Na) was calculated as a circular area with a radius of *σ* (min) to 20*σ* (max).

The clonal subrange (the spatial domain within which clonality affects the genetic structure of the population) was determined by plotting ramet and genet level pairwise kinship coefficients together with the probability of clonal identity (*F*_(r)_). The point where both correlograms merge and *F*_(r)_ is zero is the approximate distance at which clonality has no further effect on genetic structure (Alberto et al. 2005).

#### Genotypes of seedlings

During August 2009, 94 mature fruits were collected at Back‐Reef. The seeds of each fruit were placed in a small mesh bag and left to grow in flow‐through outdoor tanks (L 1.2 m × W 0.3 m × D 0.6 m) for 2 months until they had formed a well‐developed juvenile shoot with enough foliar tissue for DNA extraction. A total of 162 seedlings were obtained. DNA extraction and genotyping followed methodology of section “Population genetics”, with the addition of two microsatellite loci (TTMS‐GA72 and TTMS‐GT104; Table S3; modified from Van Dijk et al. 2007). Data were analyzed for HW disequilibrium to assess whether there was a generalized excess or deficiency of heterozygotes that would indicate the presence of outbreeding or inbreeding. The same procedure was applied to 49 seedlings from 26 fruits collected at Mid‐Lagoon in August/September 2010.

## Results

### Dispersal of seeds and pollen

Local displacement of the seeds was highly limited (Table 1, Fig. 2) and had a leptokurtic dispersal curve with only a minute proportion of the seeds traveling ≥1 m (Fig. 3). Seed dispersal was directional at the Back‐Reef, but not at Mid‐Lagoon (Table 1).

Pollen grains travelled farther than seeds. During the trials in 2005, the pollen usually reached the traps 2.4m (Mid‐Lagoon) or 4.8m (Back‐Reef) away (Table S2). The dispersal range of pollen was higher at Back‐Reef than Mid‐Lagoon (Fig. 2) and current velocities were also higher at Back‐Reef (Table S1). During the 2006 trials (with larger experimental areas), 50%, 46%, and 51% of the pollen were captured on the side of the feathers that faced the pollen release point at Mid‐Lagoon (May 25) and Back‐Reef (April 27 and May 11), respectively. Pollen grains were usually uniformly distributed in the circle (Table 2), with the exception of the trial held on April 26 2006 at Back‐Reef (Table 2). No pollen was captured in the control traps in Mid‐Lagoon and only one pollen grain was trapped out of 50 traps placed in Back‐Reef.

**Table 2:** *Thalassia testudinum*. Dispersal estimates of pollen in the Puerto Morelos Reef Lagoon (2006)

Site	Date	No traps	Design			Hodjes‐Ajne, “m”	Mean (m)	*V*_0_ (m)	*K*	AF	*N*_aPollen_ (m^2^)
			Initial distance (m)	*N*_traps_	*N*_pollen_						
Back‐Reef	26 April	120	0.4	16	67	1**	2.02	0.463	5.74**	3.8	2.76
Back‐Reef	10 May	120	0.4	17	57	5^NS^	1.15	1.719	2.94*	4	10.80
Mid‐Lagoon	25 May	100	0.2	23	71	7^NS^	0.66	0.448	5.37**	3.8	1.65

Design: distance intervals of traps (initial distance “0.2”: 0–0.2–0.4–0.8–1.4–2.4; initial distance “0.4”: 0.4–0.8–1.6–2.8–4.8–8.0 m), *N*_traps_: no traps with pollen, *N*_pollen_: total number of captured pollen grains, Hodges–Ajne: “m”‐statistic of the test for circular uniformity, Mean: mean dispersal distance, *V*_0_: Axial Variance, *K*: Kurtosis (significance was determined with paired *t*‐test), AF: Approximate area correction factor (Wright 1969), *N*_aPollen_: Neighborhood area for pollen (axial variance was divided by 2), *P < 0.5, **P < 0.001, NS: not significant.

The neighborhood area (*N*_aSeed_ + *N*_aPollen_; Tables 1 and 2) of *T. testudinum* was estimated to be 7.4 m^2^ (range 3.4–11.4 m^2^) at Back‐Reef and 1.9 m^2^ (range 1.87–1.92 m^2^) at Mid‐Lagoon.

### Population genetics

#### Clone identification

A total of 200 samples were genotyped. Eleven samples were eliminated from further analysis due to genotyping failure, resulting in 96 genotypes at Back‐Reef and 93 at Mid‐Lagoon. In total, eight microsatellite markers revealed 73 alleles at both sites combined (Table S4) with a maximum of 12 alleles observed in locus GA12 in Back‐Reef and 13 alleles of the same locus in Mid‐Lagoon.

Different MLGs most likely resulted from sexual reproduction as *p*_*gen*_ varied between 3.4 × 10^−13^ and 1.09 × 10^−7^, indicating a one in a million chance to get the same genotype at random given the local genetic diversity. Ramets with identical MLGs had a *p*_sex (fis)_ that varied between 4.67 × 10^−64^ and 1.8 × 10^−6^, indicating a one in a almost two hundred thousand that the duplication is the results of a sexual event and not asexual duplication.

After removing the duplicate genotypes, genetic differentiation between sites resulted negative (it was corrected to 0), with Mid‐Lagoon and Back‐Reef effectively being part of the same genetic population (*F*_ST_ −0.0018, *P* < 0.01). The average number of alleles per locus was 9 for Back‐Reef and 8 for Mid‐Lagoon. The average observed heterozygosity (H_o_) per locus was 0.728 (range 0.460–0.888) in Back‐Reef, and 0.710 (range 0.420–0.898) in Mid‐Lagoon. The average expected heterozygosity was 0.714 (range 0.444–0.859) and 0.695 (range 0.433–0.854) in Back‐Reef and Mid‐Lagoon, respectively. (see Table S4 for all genetic variables). All loci were in HW equilibrium and no excess or deficiency of heterozygotes was found; also no significant linkage disequilibrium was observed.

Clonal diversity (R) was 0.66 at Back‐Reef and 0.75 at Mid‐Lagoon, with respective maximal genet sizes (distance between the pairs of ramets belonging to the same genet that are furtherst away from eachother) of 19.2 m (median 7.5 m) and 10.8 m (median 4.8 m), however, genets could have been extended outside the studied plots. The clonal structure at both sites was similar and consisted of few large genets intermixed with many genetically unique ramets (Fig. 3). The ramets of the genets grew dispersed (intermingled with ramets of other genets), and the aggregation index (Ac) was 0.09 and 0.10 for Back‐Reef and Mid‐Lagoon, respectively.

#### Spatial population genetic structure

Kinship coefficients were highest at the first distance class and rapidly decreased with distance. *F*_IJ_ at the ramet level at distance class 1.5 m was 0.0367 at Back‐Reef and 0.0586 at Mid‐Lagoon and for the genet level it was 0.0354 at Back‐Reef and 0.0401 at Mid‐Lagoon.

**Figure 4:**
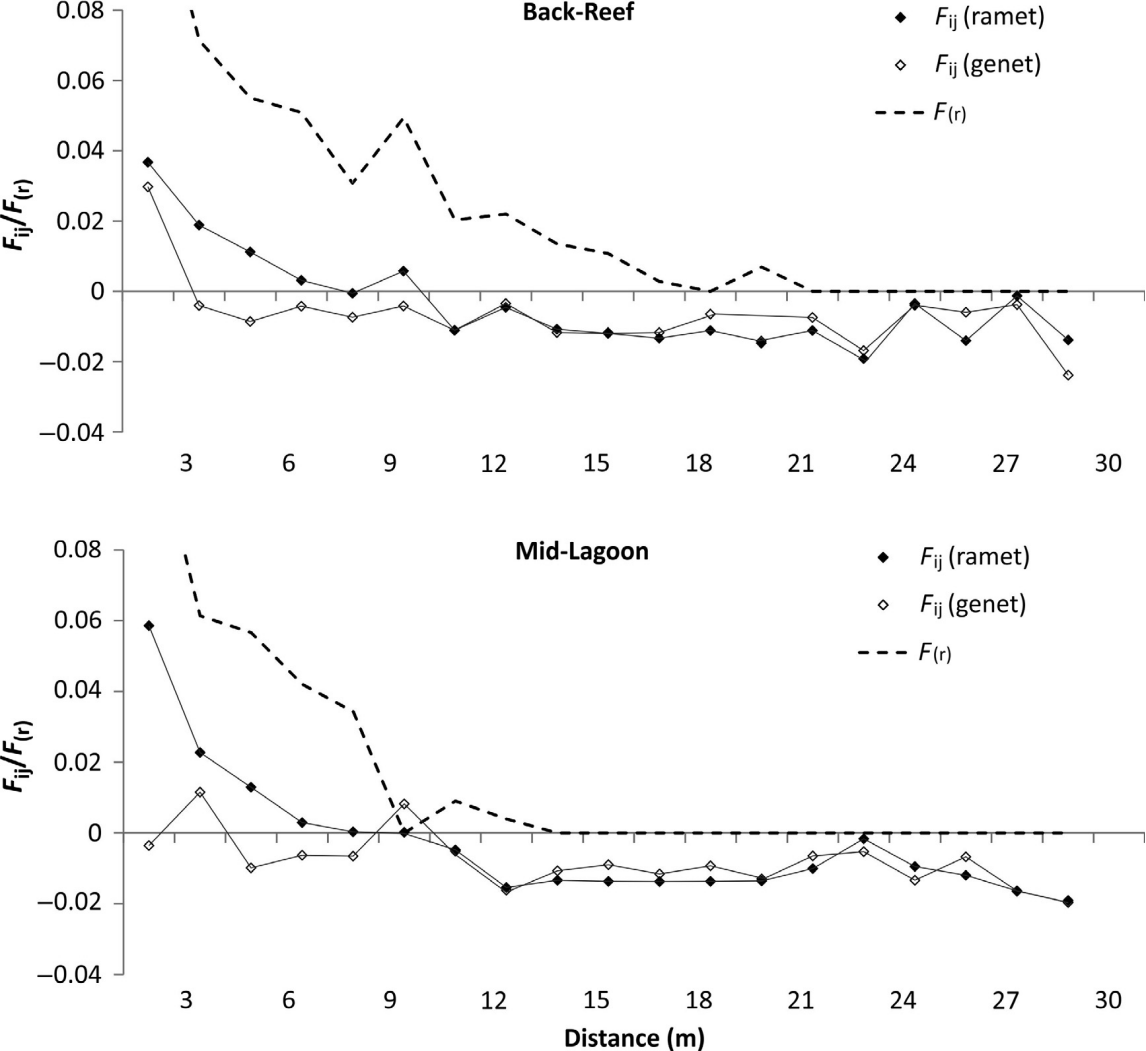
Spatial autocorrelograms of pairwise kinship coefficients (*F*_IJ_) against distance for eight microsatellite loci of ramets and genets of *Thalassia testudinum* at two study sites, together with the probability of clonal identity *F*_(r)_. Clonal subrange is determined after the first point of intersection of ramet and genet autocorrelograms and where *F*_(r)_ is zero.

Spatial autocorrelation of pairwise Fij and spatial distance between ramets was significant up until 4.5 and 6 m in Back‐Reef and Mid‐Lagoon, respectively. At the genet level, the spatial autocorrelation was significant up until 1.5 m at both sites (Fig. 4). The slope *b*_log(*P* < 0.05)_ was negative in all cases and had values of −0.0108 and −0.0136 for ramet level, and −0.0349 and −0.0391 for genet level in Back‐Reef and Mid‐Lagoon, respectively. The rates of decrease in kinship with distance (*Sp*) *Sp* were 0.0112 and 0.0144 for ramet level, and 0.0362 and 0.0407 for genet level in Back‐Reef and Mid‐Lagoon, respectively. The estimated neighborhood size (Nb) consisted of 89 and 69 individuals for ramet level, and 28 and 25 individuals for genet level in Back‐Reef and Mid‐Lagoon, respectively. The effective density of genets (D_e_) consisted of 436 genets m^−2^ at Back‐Reef, and 410 genets m^−2^ at Mid‐Lagoon. Total variance of gene dispersal ($\sigma_{g}^{2}$) was calculated for Back‐Reef and Mid‐Lagoon for ramet level (0.016 and 0.013, respectively) and genet level (0.005 in both sampling sites). The axial standard deviation of gene dispersal distance (*σ*) was 0.128 and 0.116 at ramet level, and 0.071 and 0.069 at genet level for Back‐Reef and Mid‐Lagoon, respectively. At ramet level, the neighborhood areas (Na) were 0.05–20.45 m^2^ and 0.04–16.87 m^2^ at Back‐Reef and Mid‐Lagoon, respectively. At genet level, the neighborhood areas were 0.02–6.34 m^2^ (Back‐Reef) and 0.01–5.98 m^2^ for the genet level at Mid‐Lagoon (Table 3).

**Table 3:** Parameters of spatial population genetic structure of *Thalassia testudinum* in Puerto Morelos Reef Lagoon

	*R*	*F*_(1.5)_	b_log__(*P* < 0.05)_	*Sp*	Nb	D_e_	*σ*	*σ*_g_	20*σ*_g_	Na (m)	
										Radius *σ*_g_	Radius 20*σ*_g_
Back‐Reef											
Ramet	0.66	0.0367	−0.0108	0.0112	89	436	0.016	0.128	2.551	0.05	20.45
Genet	0.66	0.0354	−0.0349	0.0362	28	436	0.005	0.071	1.420	0.02	6.34
Mid‐Lagoon											
Ramet	0.75	0.0586	−0.0136	0.0144	69	410	0.013	0.116	2.317	0.04	16.87
Genet‐Central	0.75	0.0401	−0.0391	0.0407	25	410	0.005	0.069	1.380	0.01	5.98

R clonal richness, *F*_(1.5)_ average kinship at 1.5 m distance, *b*_log_ slope of linear regression of kinship with distance, *Sp* the rate of decrease of pairwise kinship with distance, *Nb* Neighborhood size, D_e_ effective population density, *σ*_g_ axial standard deviation of gene dispersal distances derived from genetic kinship, Na neighborhood area.

Clonal subrange was 19.5 m at Back‐Reef and 10.5 m at Mid‐Lagoon (Fig. 4).

#### Genotypes of seedlings

The seedlings from Back‐Reef presented two loci (GA72 and GT77) in HW disequilibrium; locus GA72 had a deficiency of heterozygotes (*P*‐value: 0.006), but neither significant deficiency nor excess of heterozygotes was detected at GT77 (*P*‐values: 0.062 and 0.938, respectively). The seedlings from Mid‐Lagoon had two loci (GA6 and GT104) in HW disequilibrium, but we could not detect whether this could have been caused by a deficiency or excess of heterozygotes in further tests (GA6 *P*‐values: 0.080 and 0.921, respectively, and GT104 *P*‐values: 0.784 and 0.213, respectively). Overall, no general trend in global deficiency or excess of heterozygotes in the seedlings was observed at neither site (Table 4).

**Table 4:** Hardy–Weinberg statistics for seedlings of *Thalassia testudinum* in Puerto Morelos Reef Lagoon

Locus	Back‐Reef 2009				Mid‐Lagoon 2010			
	*N*	*H*_*O*_	*H*_*E*_	*P*	*N*	*H*_*O*_	*H*_*E*_	*P*
GA6	142	0.803	0.757	0.370	47	0.766	0.790	0.017*
GA8	150	0.760	0.798	0.115	42	0.857	0.800	0.829
GA12	161	0.857	0.866	0.619	47	0.894	0.871	0.222
GT77	162	0.735	0.732	0.046*	49	0.816	0.833	0.294
Th1MS	155	0.755	0.824	0.225	48	0.917	0.831	0.997
TGA39	162	0.710	0.676	0.709	49	0.592	0.669	0.110
TCT58	162	0.488	0.474	0.796	49	0.469	0.424	0.624
GGT59	161	0.578	0.550	0.498	44	0.432	0.436	0.821
GA72	108	0.565	0.663	0.023*	39	0.615	0.688	0.176
GT104	114	0.658	0.705	0.167	46	0.717	0.679	0.036*

*H*_*O*_ observed heterozygosity, *H*_*E*_ expected heterozygosity, *P* significance, * significant deviation from Hardy–Weinberg equilibrium.

## Discussion

Local gene dispersal of *Thalassia testudinum* at Puerto Morelos was extremely restricted and the estimates of the neighborhood areas based on fine‐scale genetic structure of the genet level analysis (0.02–6.34 m^2^ at Back‐Reef, 0.01–5.98 m^2^ at Mid‐Lagoon) were similar to those based on in situ dispersal of seeds and pollen (7.4 and 1.9 and m^2^ at Back‐Reef and Mid‐Lagoon, respectively). Thus, the neighborhood size derived from two independent approximations (measurements of local dispersal of sexual propagules and local genetic structure) are in agreement with each other, and mutually supportive. In situ dispersal of the seeds and pollen were measured under average hydrodynamic regimes during the reproductive season. The accordance of direct measures of pollen and seed dispersal with indirect genetic dispersal, indicates that effective local dispersal (resulting in settlement) occurs under average hydrodynamic regimes; and that incidental storms or hurricanes do not play a significant role in local gene dispersal. During storms, seeds released by fruits attached to mother plants may not travel far because their dispersal is not much affected by hydrodynamics (see below); and the pollen grains likely dilute in the water column, decreasing the probability reaching female flowers further away. The hydrodynamic regime at the Back‐Reef is rather high in comparison with many other *T. testudinum* meadows; thus we expect the area at this site to fall in the upper range of the local genetic neighborhood for this species.

Although, local dispersal is prevalent in this species, *T. testudinum* has a dual seed dispersal strategy, where ~10–15% of the fruits with seeds detaching from the maternal plant, floating to the surface to be transported over longer distances by currents and winds (Van Dijk et al. 2009). Approximately 50% of these detached fruits opened within 1 day and the seeds were deposited ~1–10 km from their site of origin (Van Dijk et al. 2009); these events of import or export of seeds, although uncommon, were likely sufficient to prevent genetic differentiation between the two study sites approximately 1.2 km apart. However, ~85–90% of the fruits open‐up in situ (Van Dijk et al. 2009), and this study indicated that the dispersal capacity of locally released seeds was very limited (0.3–0.4 m on average). Dispersal of the seeds at the Back‐Reef and Mid‐Lagoon was similar, despite the differing hydrodynamic regimes at these two sites (Table 1, Table S1). It is possible that the hydrodynamic regimes do not influence the displacement of the relatively large and heavy seeds that have settled on the bottom (where current velocities are lower; Koch et al. 2006). Alternatively seeds become immobilised when these are trapped in micro‐relieves on the rubble‐rich sediment at the Back‐Reef. Pollen grains dispersed further than the seeds; the majority was dispersed >1–2 m from their source (Table 2, Fig. 2). Current velocities were much higher in Back‐Reef than Mid‐Lagoon (Table S1), resulting in an increased dispersal at the first site (Fig. 2). Pollen grains were equally captured on either side of the traps (facing toward or away from the point of pollen release), indicating a to‐and‐from motion, which often resulted in a (near‐) return to the origin of the pollen grains in the water column, a motion pattern that has been previously described by Cox and Tomlinson (1988). Calculations of the neighborhood parameters according to Wright (1969) assume a uniform circular distribution of seed and pollen dispersal, which was not always the case in the present study (Fig. 2, Tables 1 and 2). Correction for the lack of a uniform circular distribution is difficult, but the result of such correction would be an even smaller neighborhood area than the one estimated in the present work (Crawford 1984).

The dispersal ranges of seeds and pollen with a resulting gene dispersal of 1–6 m in *T. testudinum* are very small in comparison with most terrestrial angiosperms, among which herbaceous herbs tend to have the smallest gene flow, in the order of meters (Barluenga et al. 2011), pollen transported by wind can move over large distances (>100 m – kms, Okubo and Levin 1989) and insects may disperse pollen on scales of meters to tens of meters depending on the pollinator (Barluenga et al. 2011). Many terrestrial seeds are dispersed by gravity over short distances within meters near the parental plant, but wind‐ or animal‐dispersed seeds may be transported over larger distances (Ouborg et al. 1999). Whereas the genetic neighborhood area based on water‐mediated pollen and seed dispersal of the marine angiosperm *Zostera marina* is in the upper range of that reported for terrestrial plants (>500 m^2^; Ruckelshaus 1996), that of *T. testudinum* is among the smallest (1–6 m^2^). The filamentous pollen of *Z. marina* travelled further than pollen of *T. testudinum* (Ruckelshaus 1996). In addition, contrary to findings of the present study, the dispersal variance of the seeds of *Z. marina* contributed more than twice as much as the variance of the pollen to the neighborhood area (Ruckelshaus 1996); and Manley et al. (2015) registered a mean seed dispersal distance of 130 m for this species. The cylinder‐shaped seeds of *Z. marina* are much smaller than those of *T. testudinum*, and they also go through a dormancy stage (Orth et al. 2006), most likely allowing for further transport by water currents. This difference between *Z. marina* and *T. testudinum* is further conformed by the study of spatial autocorrelation by microsatellites: a genetic neighborhood between 0.01 and 6.34 m^2^ for *T. testudinum* was estimated in this study, whereas Reusch et al. (1999a) found a genetic neighborhood size of 572 m^2^ for *Z. marina*.

The separation of male and female flowers on different plants (dioecy) enforces cross‐fertilization and in theory should enhance outcrossing and thus promote genetic variability at the cost of reduced sexual output. But separation of sexes offers no protection against sib‐mating. The small neighborhood area of *T. testudinum* in Puerto Morelos Reef Lagoon and the median size of 4.8‐7.5m of the genets suggest that the probability of pollen from a male plant encountering a sibling female is high. However, kinship values (*F*_IJ_) at the ramet level were low (between −0.0005 and 0.0586 at ramet level) and no indirect evidence of inbreeding was found (*F*_IS_ was insignificant). Genetic evidence did not support the presence of inbreeding (heterozygote deficiency) or outbreeding (excess of heterozygotes) during seedling development. Only one locus of the seedlings from Back‐Reef showed a significant heterozygote deficiency. However, incompatibility of mates may occur during pollination where selection against homozygotes would result in aborted ovules. Selection against sib‐mates at pollination would require a genetic incompatibility mechanism on the stigmas or styles of the female flowers. Pollen–stigma interactions are understudied for seagrasses and have only been reported for three temperate species, dioecious *Amphibolis antarctica*, hermaphrodite *Posidonia australis*, and monoecious *Zostera marina* (McConchie and Knox 1989). For *T. testudinum,* it is has yet to be established whether selection occurs at this level. However, aborted seed development may also be caused by pollen limitation, as was found by Van Tussenbroek et al. (2010) for this seagrass species in the study area.

Clonal growth influences the local genetic structure of all seagrasses including *T. testudinum*. In a study by Van Dijk and Van Tussenbroek (2010), it was shown that population of *T. testudinum* relied more on clonality in wave protected environments such as estuaries and mangrove lagoon than in reef lagoons, such as found in Puerto Morelos. Reef lagoons tend to show higher genotypic diversity, lower levels of kinship (smaller *r* values), smaller genets and a more complex distribution of the genets. The small clonal subrange (between 7 and 11 m) found in this study is in accordance with these findings. This clonal subrange was also low in comparison with other seagrass species, such as *Cymodocea nodosa* (20–35 m; Alberto et al. 2005) and *Posidonia oceanica* (12.7–78 m; Migliaccio et al. 2005), however, fine‐scale genetic studies should be realized for *T. testudinum* in other habitats to verify whether this reduced clonal subrange is universal for this species or that it might be influenced by type of habitat.

A skewed size distribution of the genets, with few large genets and many small clones or unique genotypes as reported in this study also coincides with the genetic structure of populations of *Z. marina* (Reusch et al. 1998), *P. oceanica* (Migliaccio et al. 2005), and *C. nodosa* (Alberto et al. 2005; Ruggiero et al. 2005a). Such size distribution may be the result of a founder effect with a few founding clones attaining large sizes, followed by repeated seedling recruitment (Alberto et al. 2005). However, in contrast with *T. testudinum*, the larger clones of *Z. marina* (Hämmerli and Reusch 2003), *C. nodosa* (Alberto et al. 2005), and *P. australis* (Sinclair et al. 2014) tended to aggregated in space to a greater or lesser degree, while the clone mates of the genets of *T. testudinum* at Puerto Morelos Reef Lagoon grew very dispersed (Ac 0.09–0.10; Fig. 3, Fig. S1), corresponding with a centrifugal “guerrilla‐type” clonal growth form, which is in accordance with the architectural models of Marbà and Duarte (1998). Seagrasses, similarly to terrestrial clonal species, exhibit a wide variation in clonal structure among species and populations, occupying a continuum between the clonal habits “phalanx” (the ramets form a tight, impenetrable assemblage) and “guerilla” (ramets are connected by long internodes and are spread out in space; Lovett‐Doust 1981; Marbà and Duarte 1998; Honnay and Jacquemyn 2008), resulting in clumped and intermingled distributions of the genets respectively. Guerilla‐type growth forms are associated with rapid resource exploitation and occupation of empty spaces but they are ineffective at exploiting resources at small (patchy) scales (Wijesinghe and Hutchings 1997), whereas the opposite phalanx strategy optimizes resource capture and occupation of space (De Kroon and Hutchings 1995). In terms of resource exploitation it may be expected that the slow‐growing, long‐lived, and climax species *T. testudinum* adopts a phalanx growth form. However, phalanx species have clumped distributions that potentially increase pollen limitation (due to increased distance between male and female genets) and sib‐mating (Charpentier 2002). Possibly, clonal climax plants such as *T. testudinum*, adopt the guerilla growth form (see also Fig. S1) to increase mate‐availability by allowing intermixing of male and female genets (Honnay and Jacquemyn 2008) and by reducing the possibility of bi‐parental inbreeding, thus increasing the possibility to encounter a nonrelated mate (Charpentier 2002; Ruggiero et al. 2005b).

In conclusion, considering the wide‐spread clonal growth combined with limited pollen and seed dispersal, no indications of bi‐parental inbreeding for *T. testudinum* in Puerto Morelos Reef Lagoon were obtained, which was contrary to our expectations. Proposed mechanisms to avoid bi‐parental inbreeding are selection against homozygotes during pollination, fecundation, or seed development (resulting in abortions of the ovules), or its guerrilla‐like growth form, which is an unusual clonal growth strategy for a climax species.

## Conflict of Interest

None declared.

## Supporting information


**Table S1.** Current velocities at Back‐Reef and Mid‐Lagoon at Puerto Morelos reef lagoon during 2005, together with wind speed and direction.
**Table S2.** *Thalassia testudinum*. Dispersal patterns of pollen with hydrodynamic parameters in the Puerto Morelos Reef Lagoon during pre‐trials in 2005.
**Table S3.** Polymorphic microsatellite markers for *Thalassia testudinum* (modified from Van Dijk et al. 2007) amplified with fluorescent markers for detection in an automated genetic analyser.
**Table S4.** Characteristics of the microsatellite loci for *Thalassia testudinum* at the two study sites after removal of replicate genotypes.
**Figue S1.** Photograph of an expanding meadow of *Thalassia testudinum*, showing long runners without aggregation, indicating a guerrilla clonal growth strategy.
