# Supplementary material for: Coping with potential bi‐parental inbreeding: limited pollen and seed dispersal and large genets in the dioecious marine angiosperm Thalassia testudinum
Source: Ecol Evol. 2016 Jul 13;6(15):5542–56. doi: 10.1002/ece3.2309 (PMC5127610; doi:10.1002/ece3.2309)

SUPPLEMENTS (**Coping with potential bi-parental inbreeding: …… *Thalassia* testu*dinum)***

Table S1. Current velocities at Back-Reef and Mid-Lagoon at Puerto Morelos reef lagoon during 2005, together with wind speed and direction. Currents were determined in a cleared patch at ~2 cm above the sea bottom, with an Argonaut ADV^®^ (SonTek, San Diego, USA), which measured single point velocities in a 0.25 cc sampling volume at intervals of 5 s for 1 h. *u* Eastern velocity component, *v* Northern velocity component*, w* vertical velocity component, *U* median current velocity*, θ* median current direction. min minimal, max maximal.

|  | **Current** | |  | |  | |  |  | |  | | |  | | **Wind** |  |
| --- | --- | --- | --- | --- | --- | --- | --- | --- | --- | --- | --- | --- | --- | --- | --- | --- |
| **Date** | ***u*_min_**  **(cm s^-1^)** | ***u*_max_**  **(cm s^-1^)** | | ***v*_min_**  **(cm s^-1^)** | | ***v*_max_**  **(cm s^-1^)** | | | ***w*_min_**  **(cm s^-1^)** | | ***w*_max_**  **(cm s^-1^)** | ***U***  **(cm s^-1^)** | | ***θ***  **(°)** | **Speed**  **(m s^-1^)** | **Direction**  **(°)** |
| **Back-Reef** | |  | |  | |  | | |  | |  |  | |  |  |  |
| 27 April | -25.4 | 19.9 | | -22.7 | | 28.4 | | | -6.5 | | 2.8 | 10.6 | | 215 | 6.5 | 300 |
| 12 May | -22.8 | 33.7 | | -20.3 | | 30.2 | | | -6.7 | | 4.3 | 7.6 | | 199 | 6.8 | 257 |
| 26 May | -27.4 | 32.3 | | -15.7 | | 25.3 | | | -4.64 | | 3.33 | 5.3 | | 181 | 4.7 | 286 |
| **Mid-Lagoon** | |  | |  | |  | | |  | |  |  | |  |  |  |
| 29 April | -6.0 | 4.7 | | -6.5 | | 8.5 | | | -5.3 | | 1.7 | 2.0 | | 146 | 6.4 | 301 |
| 16 May | -6.8 | 6.3 | | -6.9 | | 4.2 | | | -4.9 | | 2.1 | 1.6 | | 148 | 4.5 | 249 |

Table S2. *Thalassia testudinum*. Dispersal patterns of pollen with hydrodynamic parameters in the Puerto Morelos Reef Lagoon during pre-trials in 2005. Design: distance intervals of traps according to Fibonachi sequence (initial distance **0.1**: 0-0.1-0.3-0.6-1.0-1.5; initial distance **0.2**: 0-0.2-0.4-0.8-1.4-2.4; initial distance **0.4**: 0.4-0.8-1.6-2.8m), N_traps_: number of traps with pollen, Median: Median distance of pollen displacement, Max: Maximal dispersal distance, Hodges-Ajne: “m”-statistic of the test for circular uniformity. NS: not significant, NA: not applicable, nd: no data. See text for further explanation.

|  |  |  |  |  |  |  |  |
| --- | --- | --- | --- | --- | --- | --- | --- |
|  |  |  |  | **Design** | **Dispersal** | |  |
| **Site** | **Date** | **No traps** | **N_traps_** | **Distance**  **(min-max)** | **Median** | **Max** | **Hodjes-Ajne** |
|  |  |  |  | **(m)** | **(m)** | **(m)** | **“m”** |
| **Back-Reef** | 5 May | 60 | 31 | 0.1-1.5 | 0.6 | 1.5 | 11^NS^ |
| **Back-Reef** | 12 May | 68 | 39 | 0.2-2.4 | 1.4 | 2.4 | 17^NS^ |
| **Back-Reef** | 26 May | 68 | 38 | 0.2-2.4 | 0.8 | 2.4 | 17^NS^ |
| **Back-Reef** | 10 Jun | 88 | 25 | 0.4-4.8 | 1.6 | 4.8 | 7^NS^ |
| **Mid-Lagoon** | 29 Apr | 60 | 16 | 0.1-1.5 | 0.3 | 1.0 | 5^NS^ |
| **Mid-Lagoon** | 16 May | 29 | 11 | 0.2-2.4 | 0.4 | 2.4 | 4^NS^ |

Table S3. Polymorphic microsatellite markers for *Thalassia testudinum* (modified from van Dijk *et al.* 2007) amplified with fluorescent markers for detection in an automated genetic analyser. T*_m_*, melting temperature; t, X ° C touchdown - Y ° C (n) cycles; t_a_, final alignment with temperature (n) cycles; e, extension time each cycle at 72 ° C. BR: Back-Reef, ML: Mid-Lagoon.

| **Locus** | **Fluorescent label** | **Primer**  **(μM)** | **MgCl_2_**  **(mM)** | **T*_m_***  **(°C)** | **Allelic range** | **Number of alleles** |
| --- | --- | --- | --- | --- | --- | --- |
| TTMS-GA6 | VIC | 0.500 | 2.5 | t 67-57 (20), t_a_ 57 (22), e 1 min | 121-153 | 10 (BR), 7 (ML) |
| TTMS-GA8 | NED | 0.250 | 2.5 | t 65-50 (14), t_a_ 50 (20), e 1 min | 234-268 | 11 (BR), 11 (ML) |
| TTMS-GA12 | NED | 0.250 | 2 | t 65-50 (14), t_a_ 50 (20), e 1 min | 145-183 | 12 (BR), 13 (ML) |
| TTMS-GA72 | PET | 0.400 | 2 | t 68-58 (20), t_a_ 58 (30), e 1 min | 225-242 | 8 (BR), 7 (ML) |
| TTMS-GT77 | PET | 0.250 | 2 | t 65-50 (14), t_a_ 50 (20), e 45 s | 94-122 | 10 (BR), 9 (ML) |
| TTMS-GT104 | VIC | 0.400 | 2 | t 68-58 (20), t_a_ 58 (30), e 1 min | 180-191 | 5 (BR), 4 (ML) |
| TTMS-TGA39 | VIC | 0.250 | 2.5 | t 65-50 (20), t_a_ 50 (20), e 45 s | 221-233 | 5 (BR), 5 (ML) |
| TTMS-TCT58 | PET | 0.125 | 1.8 | t 65-50 (20), t_a_ 50 (20), e 45 s | 169-196 | 6 (BR), 6 (ML) |
| TTMS-GGT59 | FAM | 0.150 | 1 | 60 (43), e 45 s | 220-244 | 6 (BR), 6 (ML) |
| TTMS-Th1MS | FAM | 0.250 | 2.5 | t 65-50 (14), t_a_ 50 (20), e 45 s | 145-179 | 9 (BR), 9 (ML) |

Table S4. Characteristics of the microsatellite loci for *Thalassia testudinum* at the two study sites after removal of replicate genotypes. Na Number of alleles per locus, *H_O_* observed heterozygosity, *H_E_* expected heterozygosity, F*_IS_* inbreeding coefficient. All loci were at Hardy-Weinberg equilibrium.

|  | **Back-Reef** | | | | **Mid-Lagoon** | | | |
| --- | --- | --- | --- | --- | --- | --- | --- | --- |
| **Locus** | **Na** | ***H_O_*** | ***H_E_*** | **F*_IS_*** | **Na** | ***H_O_*** | ***H_E_*** | **F*_IS_*** |
| GA6 | 10 | 0.809 | 0.801 | 0.002 | 7 | 0.758 | 0.758 | -0.012 |
| GA8 | 11 | 0.825 | 0.805 | -0.024 | 11 | 0.724 | 0.756 | 0.042 |
| GA12 | 12 | 0.825 | 0.859 | 0.039 | 13 | 0.898 | 0.854 | -0.052 |
| GT77 | 10 | 0.857 | 0.801 | -0.070 | 9 | 0.855 | 0.800 | -0.069 |
| TGA39 | 5 | 0.682 | 0.640 | -0.066 | 5 | 0.681 | 0.670 | -0.015 |
| TCT58 | 6 | 0.476 | 0.444 | -0.072 | 6 | 0.420 | 0.433 | 0.031 |
| GGT59 | 6 | 0.460 | 0.546 | 0.159 | 7 | 0.478 | 0.485 | 0.015 |
| Th1MS | 9 | 0.888 | 0.819 | -0.086 | 9 | 0.855 | 0.800 | 0.060 |
| Mean | 9 | 0.728 | 0.714 | -0.015 | 8 | 0.710 | 0.695 | 0.000 |

Fig. S1. Photograph of an expanding meadow of *Thalassia testudinum*, showing long runners without aggregation, indicating a guerrilla clonal growth strategy. A. Bonaire, Lac Lagoon, B. Puerto Morelos, coastal fringe, C. Curacao, Spanish Water


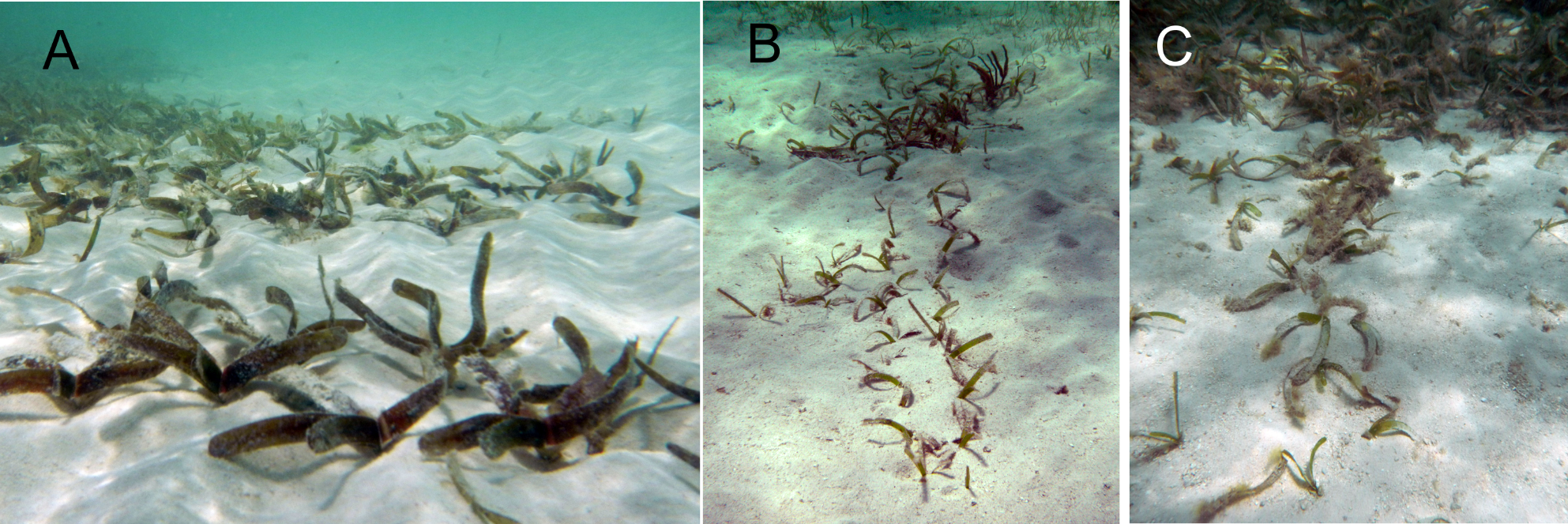

Supplement: Supplementary file 1 — Table S1. Current velocities at Back‐Reef and Mid‐Lagoon at Puerto Morelos reef lagoon during 2005, together with wind speed and direction. Table S2. Thalassia testudinum. Dispersal patterns of pollen with hydrodynamic parameters in the Puerto Morelos Reef Lagoon during pre‐trials in 2005. Table S3. Polymorphic microsatellite markers for Thalassia testudinum (modified from Van Dijk et al. 2007) amplified with fluorescent markers for detection in an automated genetic analyser. Table S4. Characteristics of the microsatellite loci for Thalassia testudinum at the two study sites after removal of replicate genotypes. Figue S1. Photograph of an expanding meadow of Thalassia testudinum, showing long runners without aggregation, indicating a guerrilla clonal growth strategy. [file ECE3-6-5542-s001.docx]
